# Prevalence and Distribution of Malocclusion Using Dewey’s Modification in Coastal Andhra Pradesh, India: A Cross-Sectional Study

**DOI:** 10.7759/cureus.42965

**Published:** 2023-08-04

**Authors:** Sarathbabu Balina, Tarakesh Karri, Venkatagiri Indugu, Rajasekhar Reddy Gade, Cheepurupalli Meher Vineesha, Chadalawada Likhita

**Affiliations:** 1 Orthodontics and Dentofacial Orthopaedics, Anil Neerukonda Institute of Dental Sciences, Visakhapatnam, IND; 2 Orthodontics and Dentofacial Orthopaedics, St. Joseph Dental College and Hospital, Eluru, IND; 3 Prosthodontics and Crown and Bridges, St. Joseph Dental College and Hospital, Eluru, IND

**Keywords:** south india, coastal andhra population, dental disease, dental care, teeth alignment, iotn index, orthodontic treatment, dewey’s modification, angle’s classification, malocclusion

## Abstract

Aim: The aim of this study is to evaluate the prevalence and distribution of malocclusion among the coastal Andhra Pradesh population in south India.

Materials and methods: The present study has a retrospective cross-sectional study design done on orthodontic records of patients who attended the Department of Orthodontics and Dentofacial Orthopedics at Lenora Institute of Dental Sciences and Hospital, Rajamahendravaram, India. Angle’s classification and Dewey’s modification were used to assess the distribution and pattern of malocclusion in patients. Statistical analysis was done using Chi-square test.

Results: The distribution of Angle’s Class I malocclusion (67%) was more common than Angle’s Class II malocclusion (30.1%) and Angle’s Class III malocclusion (2.1%). The distribution of Class II division 1 was 23.2%, whereas Class II division 2 was 2.1% and Class II subdivision was 5.6%. Gender distribution according to Angle’s classification exhibited a statistically significant difference (p-value < 0.001). Dewey’s Class I type 2 was identified as maximum with 43.6% but no statistically significant gender distribution was reported.

Conclusion: Angle's class I malocclusion was more prevalent with a distribution of 67%, followed by Class II malocclusion (30.1%) and Class III malocclusion (2.1%). A significant number of female patients were reported with Class I and Class II malocclusion whereas Class III malocclusion was predominately seen in males. It was noticed that, among all the malocclusions, Dewey’s Class I type 2 was observed to be maximum but no significant gender distribution was observed.

## Introduction

Well-aligned teeth can have an impact on the personality of the individual, along with their contribution to the health of the stomatognathic system and oral cavity. Malocclusion affects not only the health of tissues of the oral cavity but can also create social and psychological problems. It is essential to establish the pattern of occlusal disharmony in the community in order to properly direct resources towards its diagnosis and treatment planning [1].

In a particular area, the significance of any disease can be estimated by its prevalence. For any target group in a community, a systematic and well-organized dental care programme needs certain basic data, like the prevalence of the condition. There was sufficient fundamental knowledge on the occurrence of the specific condition, available in more advanced regions of the world where the fields of orthodontics and paediatric dentistry have become specializations. Such data is still lacking in developing countries like India where oral health programmes and preventive measures are far from meeting needs [2].

At present, malocclusion is the third most common dental disease after dental caries and periodontal diseases. In fact, with the reduction in the prevalence of dental caries, malocclusion is receiving more attention [3]. It has been discovered that it varies by nation, with figures ranging from 88.1% in Colombia, 62.4% in Saudi Arabia, 20-43% in India and 20-35% in the United States. So it is necessary to conduct epidemiologic research in all regions and from various geographical locations regarding malocclusion. Understanding the causes of malocclusion may also benefit from an evaluation of the prevalence rates of malocclusion in those groups. From an epidemiologic perspective, good documentation is important because it describes the range of occlusal deviations in a community where we can offer orthodontic therapy [4].

Very few studies have been conducted on the prevalence of malocclusion in patients who needs orthodontic treatment in the coast region of Andhra Pradesh in India. Thus, the aim of this study is to assess the prevalence of malocclusion and its gender distribution among the coastal Andhra population in south India.

## Materials and methods

This is a retrospective cross-sectional study done with the help of orthodontic records (study models, photographs, and case history) of patients who presented to the Department of Orthodontics at Lenora Institute of Dental Sciences, Rajamahendravaram, Andhra Pradesh, India. The records of 748 patients over the period of six months (January-June, 2022) were examined and records fulfilling the eligibility criteria were included in the study.

Inclusion and exclusion criteria

Inclusion criteria were: (i) permanent dentition present and without any deciduous tooth remaining; (ii) patients having maxillary and mandibular permanent first molars; (iii) no large restoration in the crown that might affect both shape and size. Exclusion criteria were: (i) records of patients who underwent orthodontic treatment; (ii) patients with missing or grossly decayed maxillary/mandibular first permanent molars. Examination of all the records was done by a single orthodontist and assessed at maximum intercuspation occlusal relationships.

Classification

The malocclusions were classified as given in Table 1 [5].

**Table 1:** Classification of malocclusion according to Dewey’s modification

Type		Dewey’s modification
Class I	Presence of bilateral Angle’s Class I molar relationship	Type 1 (crowded incisors)
		Type 2 (protruded maxillary incisors)
		Type 3 (anterior cross-bite)
		Type 4 (unilateral or bilateral posterior crossbite)
		Type 5 (mesial drift of molars)
Class II	Division 1 malocclusion	
	Division 2 malocclusion	
	Subdivision malocclusion (one side Class II & other side Class I)	
Class III	Presence of Class III molar relationship	Type 1 (edge-edge relationship)
		Type 2 (crowded mandibular incisors)
		Type 3 (crowded maxillary incisors, and anterior crossbite)
	Subdivision malocclusion (one side Class III & other side Class I)	

Statistical analysis

Statistical analysis was done using IBM SPSS Statistics for Windows, Version 23.0 (Released 2015; IBM Corp., Armonk, New York, United States) and included descriptive statistics and Chi-square test. The statistical significance level was fixed at 0.05.

## Results

Out of 466 patients included in the study, 312 patients were reported to have Class I malocclusion (67.0%), 144 patients to have Class II malocclusion, of which 108 patients had Class II division I (23.2%) and 10 patients had Class II division 2 (2.1%) and 26 patients had Class II subdivision (5.6%). Class III malocclusion (2.1%) was present in a total of 10 patients (Table 2).

**Table 2:** Distribution of patients according to Angle’s classification

S. No.	Type		Frequency	Percent
1	Class I		312	67
2	Class II	division 1	108	23.2
		division 2	10	2.1
		subdivision	26	5.6
3	Class III		10	2.1
	Total		466	100

**Figure 1:**
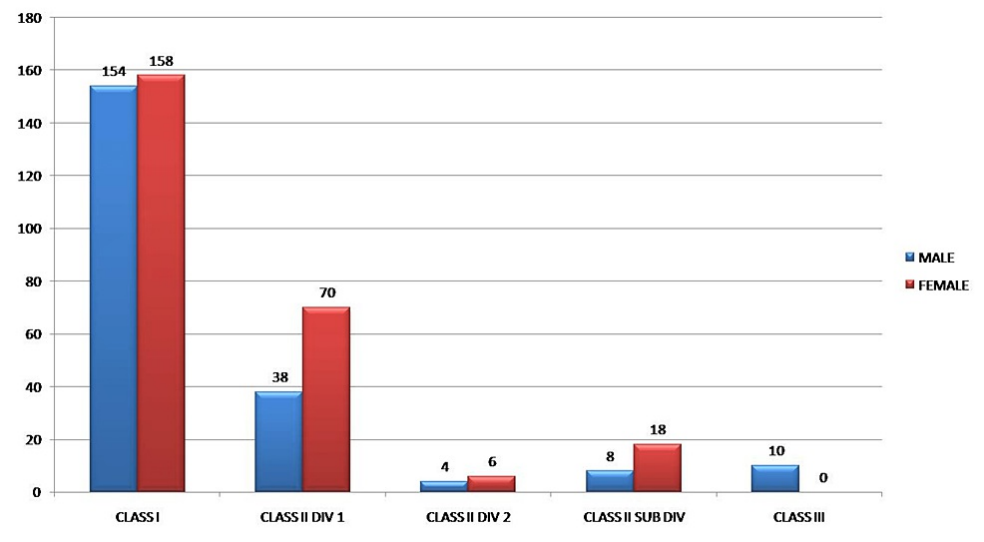
Gender distribution of patients according to Angle’s classification

Gender distribution of patients according to Angle’s classification showed a statistically significant difference (p-value < 0.001). Among 466 patients, 214 were males and 252 were females. Out of 312 Class I patients, 154 patients were males and 158 patients were females. Among 108 Class II division 1 patients, 38 were males and 70 were females. Out of 10 Class II division 2 patients, four were males and six were females and of 26 Class II subdivision patients, eight were males and 18 were females. Among 10 Class III patients, all 10 were males with no female distribution (Table 3, Figure 1)

**Table 3:** Gender distribution of patients according to Angle’s classification Statistical inference: Pearson Chi-Square = 20.819 df =  4 p-value < 0.001

S. No.	Type		Males- N (%)	Females- N (%)	Total
1	Class I		154 (49.4%)	158 (50.6%)	312
2	Class II	division 1	38 (35.2% )	70 (64.8% )	108
		division 2	4 (40% )	6 (60% )	10
		subdivision	8 (30.8% )	18 (69.2% )	26
3	Class III		10 (100%)	0 (0% )	10
	Total		214 (45.9% )	252 (54.1% )	466

**Figure 2:**
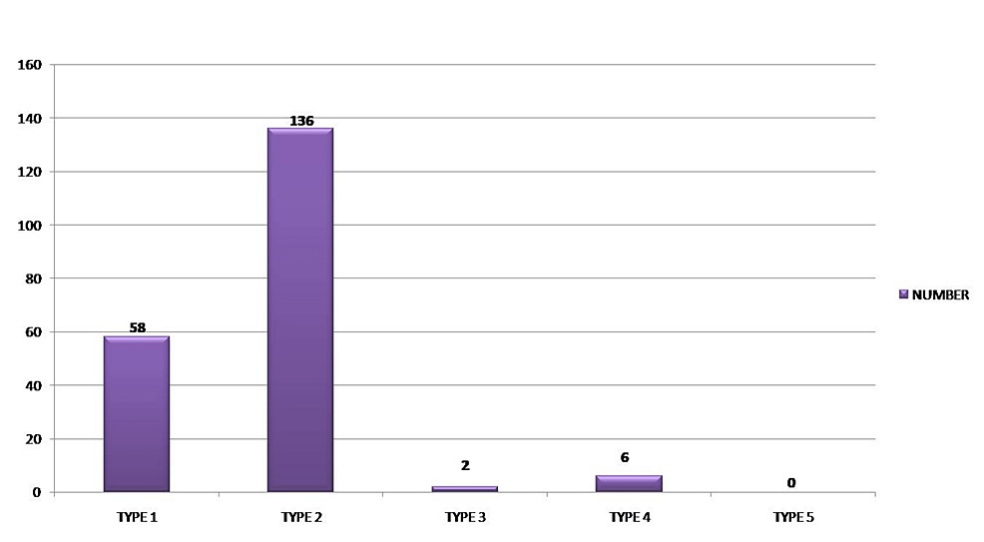
Gender distribution of patients with Angle’s class I according to Dewey’s modification

Among 312 Class I patients, 58 patients were of Dewey’s type I modification, 136 were type 2, two were type 3, six were type 4, with no type 5 modification, and 110 patients were without any Dewey’s modification. No statistically significant difference (p-value > 0.001) was observed for gender distribution in Dewey's modification. (Table 4, Figure 2)

**Table 4:** Gender distribution of patients with Angle’s class I according to Dewey’s modification Statistical inference: Pearson Chi-Square =  8.206 df =  4 p-value = 0.084

S. No.	Dewey’s modification	Males, N (%)	Females, N (%)	Total, N (%)
1	No modification	52 (47.3%)	58 (52.7%)	110 (35.3%)
2	Dewey modification 1	22 (37.9%)	36 (62.1%)	58 (18.6 %)
3	Dewey modification 2	76 (55.9%)	60 (44.1%)	136 (43.6 %)
4	Dewey modification 3	2 (100.0%)	0 (0.0%)	2 (0.6 %)
5	Dewey modification 4	2 (33.3%)	4 (66.7%)	6 (1.9 %)
6	Dewey modification 5	0	0	0 (0 %)
	Total	154 (49.4%)	158 (50.6%)	312 (100 %)

Among 10 Class III patients, two patients were of Dewey’s type I modification and eight patients were without any Dewey’s modification (Table 5).

**Table 5:** Gender distribution of patients with Angle's class III according to Dewey's modification

Sl.No	Dewey’s modification	Males, N (%)	Females, N (%)	Total, N (%)
1	Angle class III with Dewey modification 1	2 (100%)	0	2 (20%)
2	Angle class III with Dewey modification 2	0	0	0
3	Angle class III with Dewey modification 3	0	0	0
4	Angle class III with no Dewey modification	8 (100%)	0	8 (80%)
	Total	10	0	10

## Discussion

Orthodontic patient evaluation with a variety of characteristics (age, gender, and kind of malocclusion) can provide important information for organizing orthodontic treatment. Angle's Class I malocclusion (67%) was more prevalent in the current study than Angle's Class II malocclusion (30.1%) and Angle's Class III malocclusion (2.1%). Class II division 1 accounted for 23.2% whereas Class II division 2 and Class II subdivision were 2.1% and 5.6% respectively.

These findings corroborated with those of Tripathy et al., who found a greater prevalence of Angle’s Class I malocclusion (62.18%), followed by Angle’s Class II malocclusion (34.87%) and Angle’s Class III malocclusion (2.93%). Class II division 1 had a rate of 28.7% while Class II division 2 with 6.1% [6].

According to Trehan et al., Angle’s Class I malocclusion was more prevalent than Angle’s Class II division 1 malocclusion [7]. In each of the aforementioned studies, the distribution of Class II division 2 and Class III malocclusion was minimal. This is in accordance with the findings of Kharbanda et al., who revealed the prevalence of Class II division 2 malocclusion as 5.85% and of Class III malocclusion as 3.17% in his study in the north Indian population needing orthodontic treatment [8].

Among all the patients, males were 45.9% and females were 54.1%. When a comparison was made, a significant number of female patients reported malocclusion. Similar findings were reported in the study conducted by Tripathy et al., where 56.4% were females and 43.6% were males in terms of malocclusion [6]. However, Trehan et al. found no evidence of gender difference [7]. The greater number of females presenting with malocclusion may be due to esthetic considerations and psycho-social factors.

In the current study, among all the malocclusions, Dewey’s Class I type 2 was observed as maximum (43.6%) but no statistically significant gender distribution was noted. This is in accordance with the study done by Tripathy et al., in which Class I type 2 was reported as maximum with 30.9%, but a greater percentage of females were observed to have this type of malocclusion than males with significantly higher difference [6]. Devagiri V in his study found that Dewey Class I type 1 was reported as higher than type 3 but no statistically significant difference was observed among the types [9].

Data on the pattern or distribution of malocclusion alone cannot indicate a society's treatment needs. When there is no immediate worry for a dental irregularity such as diastema or tooth rotation, orthodontic treatment may not be necessary. The Index of Orthodontic Treatment Need (IOTN) is the most popular of the several indices created to prioritize malocclusion from the standpoint of the treatment [6,10-12].

In the current study, some of the patients were not categorized into Dewey’s modification because of the absence of characteristics reported in Dewey’s modified Angle’s classification. So other classifications like Ackerman & proffit might be helpful in further studies [13]. The limitations of the present study include the sample size, which was not so large. It was also unicentric as it is localized to only one population (Andhra Pradesh, India). Also, as it was a retrospective study, false negative results can be expected.

## Conclusions

The present study findings in the coastal Andhra population were as follows: Angle’s Class I malocclusion (67%) was more frequent than Angle’s Class II malocclusion (30.1%) and Class III malocclusion (2.1%). Among Class II malocclusion, Class II division 1 is more frequent than division 2 and subdivision. A significant number of female patients reported with Class I and Class II malocclusion whereas Class III malocclusion predominated in males. It was observed that, of all the malocclusions, Dewey’s Class I type 2 was identified to be having maximum frequency but no significant gender distribution was observed.
